# Severe dysphagia due to a huge epiphrenic diverticulum: long-term treatment with balloon dilation and botulinum toxin injection: a case report

**DOI:** 10.4076/1757-1626-2-7418

**Published:** 2009-06-11

**Authors:** Panagiotis Katsinelos, Grigoris Chatzimavroudis, Kostas Fasoulas, Ioannis Pilpilidis, Georgia Lazaraki, Taxiarchis Katsinelos, Dimitris Tzilves, George Germanidis, Themistoklis Vasiliadis

**Affiliations:** Department of Endoscopy and Motility Unit, “G. Gennimatas” General Hospital of ThessalonikiEthnikis Aminis 41, ThessalonikiGreece

## Abstract

We herein describe the first case of a high elderly patient with severe dysphagia in solids and liquids, caused by a huge epiphrenic diverticulum, who was treated with combined therapy of balloon dilation and botulinum toxin injection. Due to comorbid associated diseases the patient was unsuitable to withstand surgical or laparoscopic intervention. Treatment with botulinum toxin injection at the region of lower esophageal sphincter was unsuccessful. Combined therapy with balloon dilatation and botulinum toxin injection at the compressed part of esophageal lumen by the diverticulum resulted in improvement in dysphagia and malnutrition. During the long-term follow-up the patient developed symptomatic relapses, successfully treated by subsequent combined therapy resulting in longer-lasting symptom relief.

## Introduction

Epiphrenic diverticula (ED) are typically located 4 to 8 cm above the cardia, comprising about 10% of all esophageal diverticula [1]. They usually project from the right posterior wall of the esophagus and may be single or multiple. Despite ED are usually asymptomatic, especially when they are small, the enlargement of their size can produce symptoms such as dysphagia, regurgitation of undigested food, retrosternal discomfort, halitosis, and weight loss [1,2]. Reported complications include the occurrence of squamous cell carcinoma within the diverticula, hemorrhage, food impaction, bezoars formation and esophageal lumen obstruction [1-4].

We herein present the first case of a high elderly patient with severe dysphagia in solids and liquids, caused by a huge epiphrenic diverticulum, who was unsuitable for surgery due to comorbid diseases and who was treated with combined therapy of balloon dilation and botulinum toxin (BTX) injection.

## Case presentation

**Figure 1.**
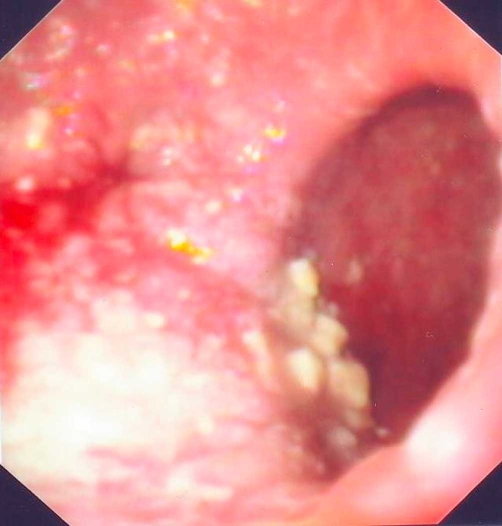
Endoscopic view of a huge epiphrenic diverticulum associated with stagnation esophagitis.

An 88-year-old man with chronic obstructive pulmonary disease and heart failure was referred to our department because he presented severe dysphagia in solid and liquid food during the last 6 months, leading to malnutrition. Clinical examination revealed an emaciated man. Laboratory data showed Hb of 11.3 g/dl (normal range: 13.5-16). Ht of 30% (normal range: 40-48%), serum albumin of 1.9 g/dl (normal range: 3.5-5) and normal liver and renal biochemistry. Esophagogram demonstrated a huge epiphrenic diverticulum in the posterior-lateral wall. Upper gastrointestinal endoscopy disclosed food debris and fluids in the esophagus. After aspiration of fluids and removal of food debris, a huge diverticulum associated with stagnation's esophagitis was demonstrated (Figure 1). The lumen of the esophagus was compressed by the diverticulum, resulting in obstruction and dysphagia. Esophageal manometry revealed findings of non-specific motility disorder.

Due to patient's associated comorbid diseases and poor general condition, surgical or laparoscopic treatment was considered unsuitable. We tried to treat the patient with BTX injection (100 IU) in the lower esophageal sphincter, with unsuccessful results. Endoscopic treatment that would combine balloon dilation of compressed by the diverticulum esophageal lumen with simultaneous BTX (100 IU) injection in the proximal aspects of narrowest area of esophageal lumen was considered as an alternative treatment that could be applied in this high-risk patient.

**Figure 2.**
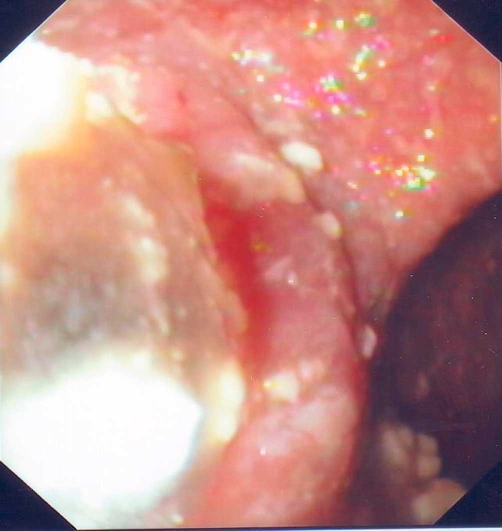
Endoscopic view showing the dilation of the compressed, by the diverticulum, esophageal lumen.

**Figure 3.**
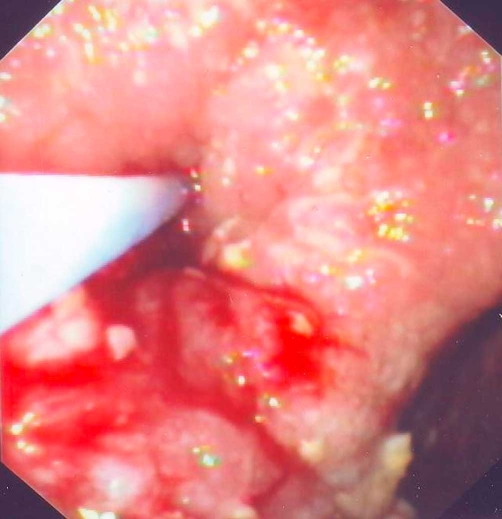
The presence of blood is due to BTX injection.

After an extensive discussion with patient's relatives about benefits and complications of our suggested treatment, a consent form was signed by them. We dilated the compressed by the diverticulum esophageal lumen (Figure 2) with a through-the-scope balloon dilator (diameter 25 mm, Microvasive, Boston Scientific, USA). The duration of dilation was 1 min and was repeated three times. The dilation was followed by BTX injection. BTX of 100 IU (Botox, Merc, Frankfurt, Germany) was dissolved in 4 ml of normal saline (25 IU/ml) immediately before injection. Injections (1 ml each) via a sclerotherapy needle were introduced in the anterior, posterior and lateral parts of the proximal narrowest area of esophageal lumen (Figure 3). The patient showed a remarkable relief of his dysphagia since the first post-procedure week, resulting in weight gain and gradual improvement of walking. The beneficial effect of combined therapy lasted 8 weeks and thereafter the therapy was repeated every 6 to 8 months during last 28 months with very good results.

## Discussion

Surgical or laparoscopic diverticulectomy combined with long esophagomyotomy is considered the treatment of choice for symptomatic patients with ED [2,3,5,6]. However, for elderly patients with comorbid conditions, such as our patient, surgical or even laparoscopic intervention is associated with significant morbidity and mortality. As a consequence we preferred to start treatment with BTX injection in the lower esophageal sphincter. Despite BTX injection has been widely used in the treatment of achalasia [7], the reported role of the use of BTX in other dyskinetic diseases of the esophagus, such as diffuse esophageal spasm, isolated LES hypertension, dysphagia due to esophageal diverticula and other specific esophageal disorders, is very limited [8,9].

There are only 3 reports on the use of BTX injection to resolve dysphagia due to midesophageal or epiphrenic diverticula. DeVault injected 80 IU (1 ml of 20 IU) through a 5 mm sclerotherapy needle into each of the four quadrants of the proximal aspect of the narrowed area distal to the diverticulum [10]. All 3 patients with midesophageal diverticula had remarkable improvement in their swallowing and remained in remission at 6-month follow-up examination. Unfortunately, there is no information on the long-term course of these patients. Pitchford and Price described a 75-year-old man with symptomatic epiphrenic diverticulum in whom they injected 100 IU of BTX into the LES, providing complete symptomatic relief [11]. Ten months later, however, the patient presented again with recurrent symptoms and underwent epiphrenic diverticulectomy. In a recent published paper we have described two elderly patients with dysphagia caused by large epiphrenic diverticula who were treated with BTX injected endoscopically at multiple sites in the region of the lower esophageal sphincter and esophageal wall near to the diverticulum, because they were unable to withstand surgical or laparoscopic intervention due to severe associated comorbid diseases [12]. Symptoms improved immediately and the beneficial effect of BTX remained for 5-6 months. During the long-term follow-up the patient developed symptomatic relapses, treated by subsequent BTX re-injections resulting in longer-lasting symptom relief.

In the patient described herein the initial use of BTX injection (100 IU) in the lower esophageal sphincter was not associated with symptom's relief. Our dilemma was to proceed to gastrostomy, enteral feeding, or to try an endoscopic treatment which combined the balloon dilation of compressed by the diverticulum esophageal lumen with simultaneous BTX (100 IU) injection in the proximal aspects of narrowest area of esophageal lumen. The relief of symptoms justified our therapeutic choice.

## Conclusions

Combined treatment of BTX injection and balloon dilation of the narrowest area of the esophagus is a simple, safe and effective method, alternative to surgery, for high elderly patients with dysphagia due to ED and severe comorbid diseases.
